# West Nile Virus Transmission by Solid Organ Transplantation and Considerations for Organ Donor Screening Practices, United States

**DOI:** 10.3201/eid2802.211697

**Published:** 2022-02

**Authors:** Raymond A. Soto, Emily McDonald, Pallavi Annambhotla, Jason O. Velez, Janeen Laven, Amanda J. Panella, Kimberly D. Machesky, Jennifer L. White, Judie Hyun, Emily Freuck, Janice Habel, David Oh, Marilyn Levi, Rick Hasz, Elling Eidbo, J. Erin Staples, Sridhar V. Basavaraju, Carolyn V. Gould

**Affiliations:** Centers for Disease Control and Prevention, Fort Collins, Colorado, USA (R.A. Soto, E. McDonald, J.O. Velez, J. Laven, A.J. Panella, J.E. Staples, C.V. Gould);; Centers for Disease Control and Prevention, Atlanta, Georgia, USA (P.D. Annambhotla, S.V. Basavaraju);; Ohio Department of Health, Columbus, Ohio, USA (K.D. Machesky);; New York Department of Health, Albany, New York, USA (J.L. White);; Maryland Department of Health, Baltimore, Maryland, USA (J. Hyun);; University of Cincinnati, Cincinnati, Ohio, USA (E. Freuck, J. Habel, D. Oh);; Health Resources and Services Administration, Rockville, Maryland, USA (M. Levi);; Association of Organ Procurement Organizations, Vienna, Virginia, USA (R. Hasz, E. Eidbo)

**Keywords:** West Nile virus, viruses, arboviruses, transmission, solid organ transplantation, donor screening, organ donor screening practices, organ procurement organization, neuroinvasive disease, United States

## Abstract

West Nile virus (WNV) is the most common domestic arbovirus in the United States. During 2018, WNV was transmitted through solid organ transplantation to 2 recipients who had neuroinvasive disease develop. Because of increased illness and death in transplant recipients, organ procurement organizations should consider screening during region-specific WNV transmission months.

West Nile virus (WNV) is the leading cause of mosquitoborne disease in the contiguous United States and is spread to humans primarily by *Culex* species mosquitoes (*1*). Transmission through solid organ transplantation (SOT) and blood transfusion was recognized during 2002 (*2**,**3*). Patients infected by SOT are at increased risk for severe disease and death, probably related to immunosuppression (*2**,**4**–**11*). In the United States, WNV screening of deceased organ donors is not mandatory and varies by organ procurement organization (OPO) (*12*). We describe WNV SOT transmission during 2018 and considerations for donor screening practices.

## The Study

A man in his 60s who had diabetes mellitus, end-stage renal disease, and hepatitis C virus infection underwent kidney transplantation during September 2018. Nine days after transplantation, he was hospitalized because of fever and progressive obtundation. Detection of serum WNV IgM prompted suspicion of donor-derived infection. The case was reported to the United Network for Organ Sharing/Organ Procurement and Transplantation Network for investigation by the ad hoc Disease Transmission Advisory Committee and subsequent referral to the Centers for Disease Control and Prevention (CDC). The patient survived with no apparent neurologic deficits.

A recipient of a liver from the same donor was a man in his 30s who had alcoholism, hepatitis C virus infection, and cirrhosis. Sixteen days after transplantation, he became febrile and was hospitalized. He survived but had mild encephalopathy and peripheral neuropathy ≈1 month after transplantation.

The organ donor was a woman in her 20s who had a history of intravenous drug use and was found at home during September in cardiopulmonary arrest attributed to drug overdose. She was resuscitated but later declared brain dead. The left kidney and liver were recovered for transplantation. Interviews of family members yielded discrepant reports about preceding symptoms. In organ donor’s state of residence, WNV human and equine disease cases, viremic blood donors, and mosquito infection rates were increased during 2018 compared with previous years.

Organ donor and recipient specimens were tested by state public health and commercial laboratories for WNV RNA by using reverse transcription PCR (RT-PCR) and for WNV IgM and IgG by using enzyme immunoassay. Additional WNV testing was performed at CDC by using RT-PCR, microsphere immunoassay for IgM, and plaque reduction neutralization testing, as described (*13**,**14*).

We detected WNV IgM and IgG in cerebrospinal fluid from the kidney recipient, and WNV RNA in serum, blood, and urine collected 18 days after transplantation. Whole blood collected 24 days after transplantation still had detectable viremia. Pretransplant specimens were not tested. Serum collected from the liver recipient 18 days after transplantation had WNV IgM and neutralizing antibodies, but no detectable WNV RNA; WNV IgM was also detected in cerebrospinal fluid. No WNV RNA, IgM, or neutralizing antibodies were detected in pretransplant serum from the liver recipient. Donor plasma and serum collected on hospital day 3 had WNV RNA and no detectable IgM or neutralizing antibodies (Table 1).

**Table 1:** West Nile virus test results for organ donor and recipients, United States*

Person	Specimen	DRT	RNA	IgM	Neutralizing antibodies (titer)†	IgG
Organ donor	Plasma	−2	Detected			
	Serum	−2	Detected	ND	ND	
Liver recipient	Serum	0	ND	ND	ND	
	Serum	18	ND	Detected	Detected (1;2,560)	
	CSF	22		Detected		
	Serum	35	ND	Detected		
Kidney recipient	Serum	15		Detected		ND
	CSF	18		Detected		Detected
	Serum	18	Detected	Detected		
	Whole blood	18	Detected			
	Urine	18	Detected			
	Serum	25	ND	Detected		
	Whole blood	25	Detected			

*Blank cells indicate testing not performed. CSF, cerebrospinal fluid; DRT, day relative to transplantation; ND, not detected. †90% plaque reduction neutralization test result <10 is considered negative.

To determine whether WNV was transmitted to the donor through blood products, the blood collection organization initiated a trace-back investigation. Remaining co-components from index donations were retrieved and quarantined, and blood donors were contacted to provide follow-up serum specimens. The organ donor received 7 blood products from 19 donors during the terminal hospitalization: 3 units of pooled cryoprecipitate (each from 5 donors), 3 units of fresh frozen plasma, and 1 unit of packed erythrocytes. All donations were screened for WNV by using minipool-nucleic acid testing (MP-NAT) before transfusion. All units of cryoprecipitate and plasma were transfused before WNV RNA-positive specimens were collected. Of 19 blood donors, 5 (26%) had no follow-up samples or co-components tested. Fourteen (74%) donors provided follow-up serum samples, including 2 who had plasma co-components from the same unit of pooled cryoprecipitate available for retrieval. All follow-up serum samples and co-components were negative for WNV IgM and neutralizing antibodies. RT-PCR was not performed for co-components because no WNV neutralizing antibodies were detected in follow-up serum samples.

## Conclusions

Less than 1% of the general population have neuroinvasive disease develop after being bitten by an infected mosquito. In contrast, we found that patients infected by SOT have high rates of severe disease and death. In previous reports with available data, 14 (67%) infected recipients had neuroinvasive disease develop and 6 (29%) died (Table 2). Both patients in our study had neuroinvasive disease develop but survived.

**Table 2:** Reported WNV solid organ transplant transmission events, United States, 2002–2013*

Date	Location	Organ donor					Organ recipient outcomes			
		WNV screening	Archived serum sample test result		Likely/proven source of infection		No. infected/ total	No. NID/no. infected	No. deaths reported	Ref
			RNA	IgM/IgG						
2002 Aug	Georgia	No	Detected	ND/NR	Blood transfusion		4/4	3/4	1	(*2*)
2005 Aug	New York, Pennsylvania	No	ND	Detected/ detected	Mosquito		3/4	2/3	2	(*4*)
2008 Sep	Louisiana	No	ND	ND/ND	Blood transfusion		1/1	1/1	0	(*5*)
2009 Sep	Italy	No	Detected	NR	Mosquito		1/1	0/1	0	(*8*)
	California	No	Detected	ND/ND	Mosquito		1/1	1/1	0	(*10*)
	Texas	No	Detected	Detected/ equivocal	Mosquito		1/3	1/1	0	CDC, unpub. data
2010 Oct	California	No	Detected	ND/detected	Mosquito		2/3	1/2	1	(*9*)
2011 Aug	Italy	NAT negative	ND	Detected/ detected	Mosquito		4/5	2/4	0	(*7*)
2011, early fall	California	No	ND (detected in spleen and lymph node)	Detected/ detected (neutralizing antibodies also detected, titer 1:160)	Mosquito		4/4	3/4	2	(*11*)
2013	NR	NR	NR	NR	NR		NR/3	NR	0	(*6*)

*CDC, Centers for Disease Control and Prevention; ND, not detected; NAT, nucleic acid test; NID, neuroinvasive disease; NR, testing not reported; ref, reference; WNV, West Nile virus.

Because blood transfusion has been implicated in organ donor infection (*2**,**5*), we conducted an extensive blood trace-back investigation. Although WNV-breakthrough transfusion transmission has been rare since routine blood donation screening was implemented in 2003, most cases have been associated with MP-NAT screening, which is less sensitive than individual donation-NAT; MP-NAT was used to test blood donations in this investigation (*15*). Although we could not definitively rule out blood transfusion as the source, the organ donor was probably infected through mosquitoes because widespread WNV transmission was occurring in the area of residence of the donor.

The organ donor did not undergo WNV screening but was found to have WNV viremia on testing of preprocurement specimens. Of 10 reports of SOT transmission, 8 occurred with no WNV donor screening (5 with RNA detected in archived serum samples), 1 occurred despite negative results for donor NAT screening (RNA also not detected in archived serum samples), and 1 did not report screening (Table 2). RNA might not be detected in infected donors because of hemodilution from transfusions and resuscitation efforts, undetectable viremia early or late in infection, assay detection limitations, or viral persistence in organs after resolution of viremia (*11*).

OPOs conduct extensive testing for infectious diseases, with direction and guidance from boards, medical advisory committees, and medical directors. For living donors, protocols are required for identifying and testing donors at risk for transmissible seasonal or regional endemic diseases, such as WNV disease. In contrast, there is no national policy requiring WNV screening of deceased organ donors. Limitations of screening include costs and logistical challenges of timely testing, false-negative screening results (*7**,**12*), and false-positive results that could lead to organ wasting and delays in lifesaving organ transplantation. A survey during 2008 found that 11 (19%) of 58 US OPOs performed WNV NAT screening of deceased potential organ donors (*12*). A follow-up survey conducted during 2019–2020 found that 16 (35%) of 46 OPOs performed WNV NAT screening, 5 in combination with IgM assays. Most OPOs performed year-round screening, and only 1 conducted seasonal screening (*15*).

During 2009‒2018, a total of 89% of WNV disease cases reported nationally had illness onset during July–September (*1*). Furthermore, all WNV SOT transmission events, including the ones we report here, occurred during August–October (Table 2). Therefore, seasonal screening is likely to capture most cases and be more cost-effective than year-round testing. Seasonal screening could also improve the positive predictive value of screening results, reducing the risk for false-positive results. Although spatiotemporal variability in WNV disease incidence is high, OPOs in regions that have consistently high rates of WNV disease might have a more favorable cost-benefit ratio with screening (https://www.cdc.gov/westnile/statsmaps/cumMapsData.html). A triggering strategy using local blood donation screening and working with local or state health departments to determine times of increased virus circulation could also be considered. For a screening strategy to be successful, OPOs would need systems in place for timely test results to prevent allocation of organs from WNV-positive donors.

Given the high risk for severe WNV disease in SOT recipients, OPOs should consider the feasibility of WNV screening in organ donors, at least during months associated with regional WNV transmission. Transplant programs should also continue to inform organ recipients and their families about the possibility of infectious diseases being transmitted by organ transplantation.
